# Enhanced cultivation of chicken primordial germ cells

**DOI:** 10.1038/s41598-023-39536-1

**Published:** 2023-07-29

**Authors:** Nima Dehdilani, Sara Yousefi Taemeh, Sylvie Rival-Gervier, Guillaume Montillet, Clémence Kress, Christian Jean, Lena Goshayeshi, Hesam Dehghani, Bertrand Pain

**Affiliations:** 1 Stem Cell Biology and Regenerative Medicine Research Group, Research Institute of Biotechnology, Ferdowsi University of Mashhad, Mashhad, Iran; 2 Stem Cell and Brain Research Institute, University of Lyon, Université Lyon 1, INSERM, INRAE, U1208, USC1361, 69500 Bron, France; 3 Division of Biotechnology, Faculty of Veterinary Medicine, Ferdowsi University of Mashhad, Mashhad, Iran; 4 Department of Basic Sciences, Faculty of Veterinary Medicine, Ferdowsi University of Mashhad, Mashhad, Iran

**Keywords:** Stem cells, Biotechnology

## Abstract

The cultivation and expansion of chicken primordial germ cells (cPGCs) are of critical importance for both biotechnological applications and the management of poultry genetic biodiversity. The feeder-free culture system has become the most popular approach for the cultivation and expansion of cPGCs. However, despite some success in the cultivation of cPGCs, the reproducibility of culture conditions across different laboratories remains a challenge. This study aimed to compare two defined and enriched media for the growth of cPGCs originating from the Hubbard JA57 broiler. To this end, cPGCs were isolated from the embryonic blood of Hamburger–Hamilton (HH) stages 14–16 and cultured at various time points. The Growth properties and characteristics of these cells were evaluated in two different culture conditions (the defined or enriched medium) and their migratory properties were assessed after genetic engineering and injection into the vasculature of 2.5-day-old chicken embryos. The main finding of this study was that the use of an enriched medium (the defined medium with Knock-Out Serum Replacement; KOSR) resulted in improved growth properties of cPGCs originating from the Hubbard JA57 broiler compared to a defined medium. The ability to cultivate and expand cPGCs is crucial for the generation of both genetically engineered birds and breeds of interest from local or commercial origins. Therefore, these results highlight the importance of choosing an appropriate culture medium for cPGCs growth and expansion.

## Introduction

Establishing the long-term culture of chicken primordial germ cells (cPGCs) is crucial for making transgenic lines, for the production of recombinant proteins in the egg white, and for biodiversity management^1^. However, this process is lengthy, taking several weeks before cPGCs are ready for genetic manipulation and the generation of genetically-engineered birds^2–4^.

The maintenance of pluripotency, germ-line competency, genomic stability, and cellular homeostasis are crucial for the primary cultivation and long-term propagation of stem cells in vitro^5^. Accordingly, the in vitro culture of unipotent stem cells, such as cPGCs, requires special culture conditions. Several culture systems, growth factors, and medium supplements have been employed to achieve these goals. In feeder-dependent culture systems, some of the required growth factors and supplements are provided by the feeder layer^2^. The cultivation and propagation of cPGCs have been achieved using three types of feeder-dependent systems, using BRL cells^2,6^, STO cells^2^, and CEF cells^7,8^. However, cross-contaminations are a potential risk with feeder-dependent systems, and the preparation of feeder layers is time-consuming and laborious.

In feeder-free cultures, commercial additives are added to the media^9–11^. In recent years, the 2-dimensional feeder-free culture of circulating cPGCs proposed by McGrew and colleagues has gained more attention for the cultivation and expansion of cPGCs^9^. They demonstrated that insulin (provided by the B27 supplement), FGF2, Activin A, and OvoTransferrin (the so-called FA-OT medium) could support the self-renewal and clonal growth of circulating cPGCs under permissive osmolality. This defined medium has been successfully used by other research groups with some modifications^10,12^. The maintenance of germ cell developmental potency and stemness, as well as the survival and proliferation of cPGCs, require defined growth factors such as FGF2, Activin A, and BMP4^9,13,14^. Previously, human SCF^2^, chicken SCF^15^, human IGF-1^9^, and mouse LIF^16^ were used to maintain pluripotency and germ cell developmental potency of cPGCs in the feeder-dependent system. Additionally, the in vitro culture of cPGCs can be improved through the use of various media supplements, including B27^9,10,12^, ITS (Insulin-Transferrin-Selenium)^10^, KOSR^7,8^, BRL cell-conditioned medium^2,6^, chicken embryonic extracts^7,8^, OT^9^, and ovalbumin^9^. The addition of fetal bovine serum (FBS) or chicken serum (CS) can also improve the supportiveness of the media, although the use of high-serum conditions (feeder-dependent systems) or low-serum conditions (feeder-free systems) can vary among studies^2,6,8^. In recent years, defined FA-OT or undefined FA-CS (FGF, Activin, chicken serum) medium has been widely used for this purpose^9,10,12^. Although the defined FA-ITS medium was also used, its results were less unsatisfactory compared to FA-OT or FA-CS^10^.

The maintenance of long-term germ-cell developmental competency in both male and female cPGCs is crucial, especially for the in vitro preservation of cPGCs for biotechnological, bio-banking and bio-diversity applications. Also, in vitro preservation of genetically-manipulated cPGCs for transgenesis applications is vital. A robust culture medium must mimic the in vivo growth requirements of cPGCs during the long-term culture by providing adequate growth factors and supplements. It has been noted that a portion of the cPGC population even in clonally derived cell lines, cultured on feeder layers, progressively lose their germ line features and differentiate^2^. In feeder-free culture, we can also observe morphological alteration of cPGCs attached to the culture dish (data not shown). This suggests that during the long-term culture, the germ-cell features of cPGCs may gradually fade. It has been reported that prolonged culture of embryonic or mesenchymal stem cells in well-defined media leads to impaired properties, aneuploidy, and DNA hypomethylation^5,17^. Thus, we reasoned that the expression status of stem cell-specific and germ cell-specific markers might be helpful for the evaluation of cPGCs and to identify whether they have kept their germ cell features in long-term culture. The expression of stem cell-specific markers such as SSEA1^18^ and germ cell-specific markers including EMA1^18^, as well as pluripotency transcription factors such as NANOG and POU5F3/OCT4^19^, has not been evaluated in the long-term cultured cPGCs at different time points. Furthermore, the expression levels of these markers and transcripts have not been compared in cPGCs cultured with different culture media. Although some long-term cultured cPGCs (> 280 days) retain their migration ability toward the chicken embryonic gonads (at HH stage 26–28)^2^, there is very little information available on the germinal status of all cPGCs in long-term culture.

The presence of undefined serum components can lead to the development of heterogeneous stem cell populations and a gradual loss of pluripotency^20^. Prolonged culture of murine embryonic stem cells (ESCs) in a defined serum-free medium has been shown to result in impaired features compared to early passages where the cells maintain their normal physiological characteristics^21,22^. Conversely, lipids have been shown to improve stem cell pluripotency and self-renewal^23–25^. AlbuMAX is a lipid-rich albumin purified from bovine plasma using a chromatographic separation method^26^. It has low lot-to-lot variability compared to serum and retains its natural lipids, making it a suitable replacement for serum in media formulation^25^. AlbuMAX has been shown to prevent differentiation and stimulate self-renewal of ESCs^25^, enhance fatty acid metabolism, facilitate the accumulation of lipid droplets, improve morphological features, and maintain full stemness of ESCs^27^.

KOSR contains insulin, human transferrin, and lipid-rich albumin, along with other essential components such as amino acids, vitamins, antioxidants, and trace elements^25^. The use of lipid-rich albumin-based medium such as AlbuMAX-containing chemically-defined medium (2i/LIF) has been shown to enhance self-renewal^25^, genomic stability, developmental potency^5^, stem cell proliferation, and accumulation of lipid droplets^27^ in stem cells. For example, the addition of AlbuMAX to 2i/LIF medium has been found to maintain pluripotency and full potency in the long-term culture of murine ESCs. Although the 2i/LIF medium keeps murine ESCs in a homogenous naïve state, prolonged culture using only that may lead to aneuploidy and DNA hypomethylation that impairs their developmental potential. However, AlbuMAX prevents X chromosome loss in female ESCs and supports their derivation and culture toward full potency.

In this study, we aimed to improve the cultivation, derivation, and expansion of cPGCs using a medium that contains KOSR, primarily consisting of lipid-rich albumin. The results showed that the KOSR-enriched medium not only enhanced and maintained the germinal features and self-renewal of cPGCs in long-term culture but also accelerated the proliferation rate compared to the defined medium without KOSR. Our findings demonstrate that the enriched medium is beneficial for both male and female cPGCs in terms of cultivation, derivation, expansion, and migration potency.

## Materials and methods

### Animal experiments

Fertilized eggs and recipient embryos were provided by local breeders and cPGCs were obtained from 2.5-day-old chicken embryos. The use of these cells and 6-day-old embryos did not require any ethical approval as they were acquired from an age before the completion of one-third of incubation time. The study is reported in accordance with the ARRIVE guidelines.

### cPGC culture media

**Figure 1:**
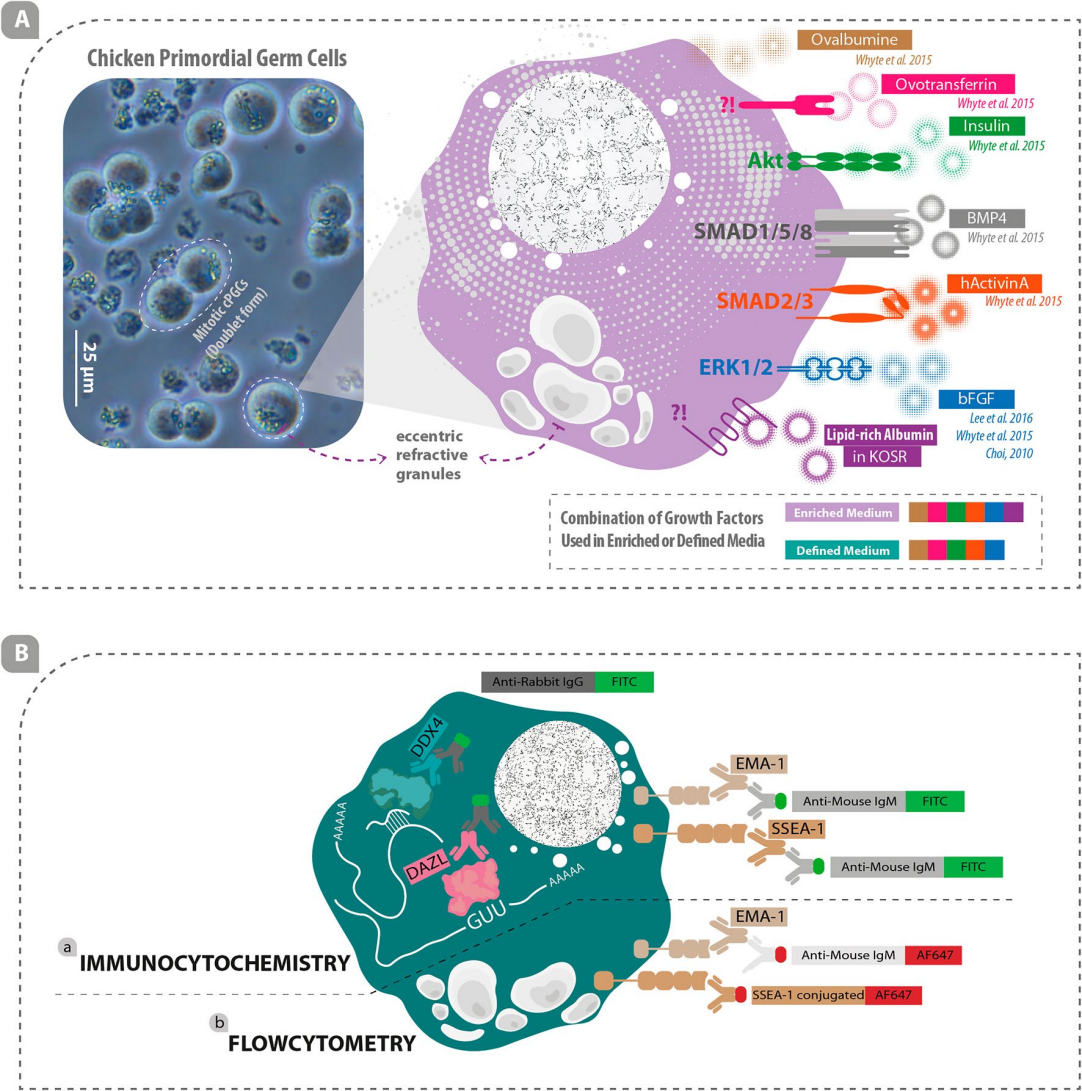
Real and schematic depiction of cPGCs, growth factors involved in cPGCs survival and proliferation in-vitro, and stem-cell/germ-cell specific markers in cPGCs. (**A**) 120-day-old cPGCs culture with lots of doublet cells and prominent eccentric refractive granules (left) and several important growth factors involved in the survival and proliferation of cPGCs are illustrated (right). (**B**-a) Immunocytochemistry was performed using primary antibodies against DDX4 and DAZL proteins as well as EMA1 and SSEA1 cell surface markers. Conjugated secondary antibodies were used to label the primary antibodies. (**B**-b) Flow cytometry was performed using primary EMA1 antibody which was labeled using conjugated secondary antibody and conjugated primary antibody was used for detecting SSEA1 cell surface marker. (IgG: Immunoglobulin G; IgM: Immunoglobulin M; FITC: Fluorescein isothiocyanate; AF647: Alexa Fluor 647).

**Figure 2:**
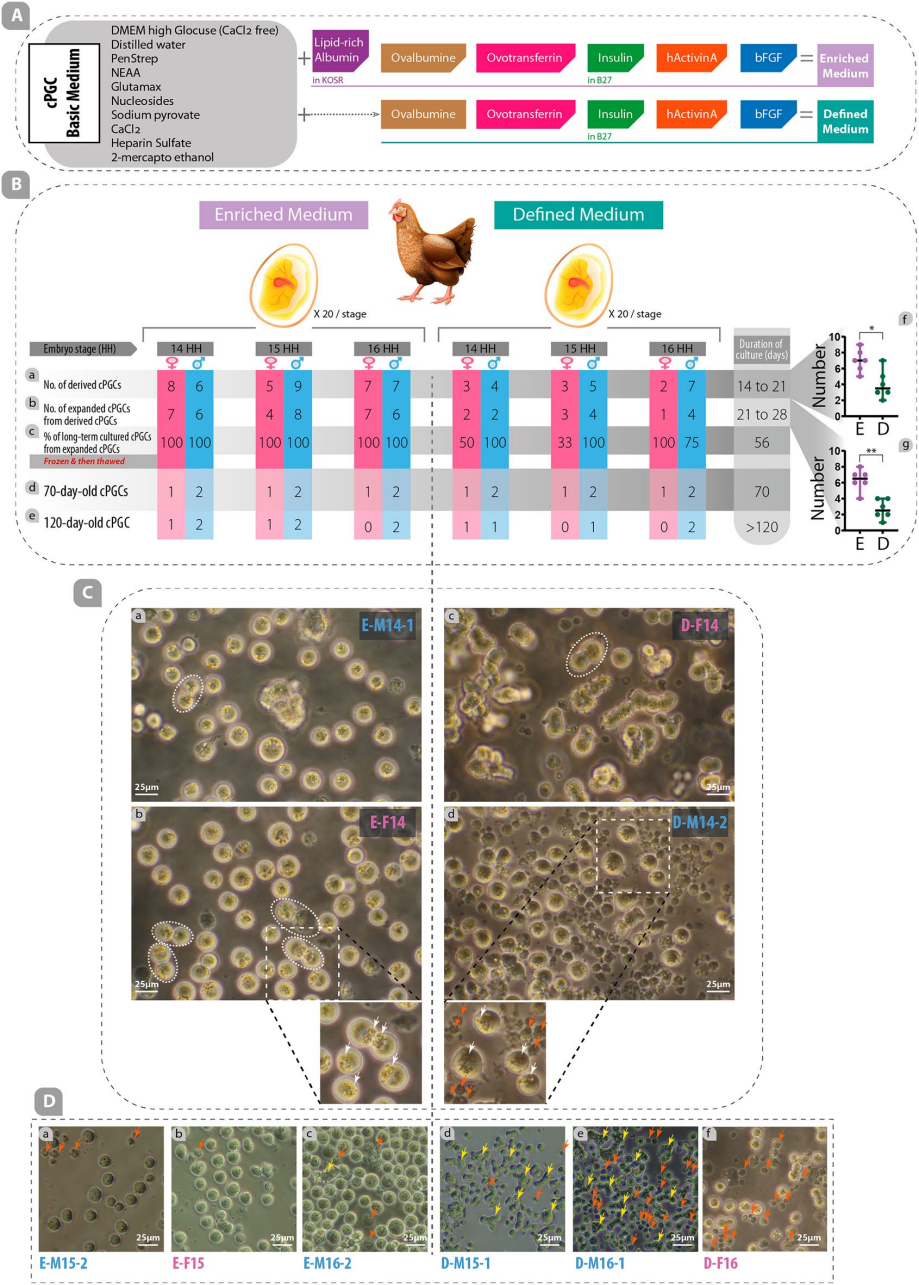
Enriched medium improves derivation, expansion, long-term culture, and proliferation rates of cPGCs. (**A**) Components of the cPGC basic medium were supplemented with several types of growth factors to make an enriched medium (specifically containing lipid-rich albumin provided by KOSR) or defined medium (without KOSR). (**B**) The rate of derived (a), expanded (b), long-term cultured (c), 70-day-old (d), and 120-day-old (e) cPGCs were compared in enriched and defined media. The number of derived (f) and expanded (g) cPGCs in the enriched medium were compared with those in the defined medium. (**C**) The specific features of cPGCs cultured in enriched and defined media were compared. The oval dash lines indicate the doublet form of cPGCs (a, b, c). In the insets, the eccentric refractive granules (white arrows) and dead cells (red arrows) are depicted (E: enriched, D: defined, M: male, F: female, 14,15,16: HH stages). (**D**) Comparison of the number of dead (red arrows) and clumpy (yellow arrows) cells in different cell lines of cPGCs cultured in enriched and defined media. *: p < 0.05, **: p < 0.01 are statistically significant.

In this study, both enriched and defined media were used to cultivate, propagate, and maintain cPGCs in long-term culture (Figs. 1A, 2A). The cPGC basic medium was prepared by diluting the DMEM (high glucose, no glutamine, no calcium; Cat#: 21068028) medium to a final osmolarity of 250 mOsm (by mixing 700 ul medium and 210 ul distilled water). This medium was supplemented with 2.0 mM GlutaMax, 1X Non-Essential Amino Acid (NEAA), 1X Penicillin/Streptomycin (Pen/Strep), 1 mM ß-mercaptoethanol, 1X nucleosides, 0.4 mM pyruvate, 0.1 mg/mL sodium heparin (Sigma), and 0.15 mM CaCl_2_ (Fig. 2A). To make the defined medium, 0.2% ovalbumin (Sigma), 10ug/mL ovotransferrin (Sigma), 1X B-27 supplement, 30 ng/mL hActivin A (Peprotech; E.Coli derived, Cat#: 120-14E), and 5 ng/mL bFGF (Peprotech; E.coli derived, Cat#: 100-18B) were added to the cPGC basic medium (Fig. 2A). By the addition of the following supplements to the cPGC basic medium, an enriched medium was made: 1% KOSR that primarily contains lipid-rich albumin, 0.2% ovalbumin (Sigma), 10ug/mL ovotransferrin (Sigma), 1X B-27 supplement, 30 ng/mL hActivin A (Peprotech; E.Coli derived), and 5 ng/mL bFGF (Peprotech; E.Coli derived) (Fig. 2A). All components used in this study were obtained from Thermo Fisher Scientific, USA, unless specified otherwise.

### Cultivation, derivation, expansion, and long-term culture of cPGCs in enriched and defined media

Embryonic blood was isolated from the dorsal aorta of fertile Hubbard JA57 broiler eggs at three different developmental stages (HH stage 14, 15, and 16; see details in supplementary Fig. S1). Samples were taken to determine the sex of the embryos. To start the primary culture, a minimum of 1 µL of embryonic blood was placed directly into 100 µL of enriched or defined media in 96-well plates. cPGCs appeared in the primary culture of embryonic blood between days 14 to 21 (Derived cPGCs; Fig. 2B-a). During this time blood cells were completely lysed and disappeared. The derived cPGCs were transferred to the 48-well plate for expansion from day 21 to 28 (Expanded cPGCs; Fig. 2B-b), and then to 24-well plates for further proliferation and long-term culture (Long-term cultured cPGCs; Fig. 2B-c). After 56 days of culture, long-term cultured cPGCs were frozen at a density of 0.5–1 × 10^6^ cells per cryovial. After three days, two male and one female cPGCs were thawed and re-cultured, and evaluated for proliferation rate. 70-day-old cPGCs (Fig. 2B-d) and 120-day-old cPGCs (Fig. 2B-e) were used for promoter assay, immunocytochemistry, flow cytometry, and qPCR. In this study, an established cell line was defined by the duration of the culture of more than 120 days (120-day-old cPGCs; Fig. 2B-e).

### Proliferation assay

Two 60-day-old male and one female cPGCs from each isolate were cultured in enriched and defined media at a density of 2 × 10^3^ cells per well of a 24-well plate (Day 0: 2000 cells/well were cultured). The cells were counted every other day from day 0 to day 10 (Supplementary Fig. S2A, B, C). To perform the assay, 10 µL of cPGCs were taken from each well plate at each time-point, stained with Trypan Blue dye (Thermo Fischer, USA), loaded onto a hemocytometer, and counted. The medium was changed every two days during the experiment.

### Analysis of germ cell- and stem cell-specific markers by immunocytochemistry

EMA1, a germ cell-specific cell surface marker, and DAZL/DDX4, germ cell-specific cytoplasmic markers, as well as SSEA1, a stem cell-specific cell surface marker, were labeled using specific primary antibodies (Anti-SSEA1 and Anti-EMA1 primary antibodies were purchased from DSHB, USA; Anti-DAZL and Anti-DDX4 primary antibodies were purchased from Abcam, USA and Biotem, France, respectively) (Fig. 1B-a). Anti-mouse IgM FITC (JIR, USA) was used as a secondary antibody to detect the Anti-SSEA1/Anti-EMA1 primary antibody (Fig. 1B-a), and anti-rabbit IgG FITC (JIR, USA) was used as a secondary antibody to detect the Anti-DAZL/ Anti-DDX4 primary antibodies (Fig. 1B-a).

For each group, 5 × 10^4^ cells were washed twice with 2% FBS-DPBS (Dulbecco's phosphate-buffered saline) and diluted in 100 μL of cold 1% BSA (bovine serum albumin)-DPBS. Slides with 2 spots and filters were placed into the cytospin’s slots with the cardboard filters facing the center of the cytospin (Thermo Fisher Scientific, USA). 100 μL of each sample was quickly aliquoted into the wells of the cytospin, and cells were cytospun at 500 rpm for 5 min. The filters were removed from the slides without contacting the cells attached to the slides. To check the morphology of cells, each slide was examined under a microscope. All the slides were then fixed with 100 μL of 4% paraformaldehyde (Thermo Fisher Scientific, USA) for 10 min. For DAZL/DDX4 labeling, the slides were treated for 15 min at room temperature with 0.5% triton X-100. The slides were washed twice with cold 1% BSA-DPBS for 5 min and then were incubated in cold 1% BSA-DPBS for 30 min. Each primary antibody, prepared in 2% BSA with appropriate dilution (0.25 µg/mL for Anti-SSEA1; 0.5 µg/mL for Anti-EMA1; 1:2000 for Anti-DDX4; 1:500 for Anti-DAZL), was poured on the slides and incubated overnight in a 4 °C wet chamber. The next day, each slide was washed twice with cold 1% BSA-DPBS for 5 min. The appropriate secondary antibody (1:500 Anti-Mouse IgM-FITC; 1:500 Anti-Rabbit IgG-FITC) was added and incubated in a 4 °C wet chamber for 1 h. The slides were washed twice with cold 1% BSA-DPBS for 5 min. Slides corresponding to EMA1 and SSEA1 were stained with DAPI for nucleus staining. Slides corresponding to DAZL and DDX4 were stained with 1μM TO-PRO-3 (Thermo Fisher Scientific, USA) for nucleus staining. Then all slides were mounted with ProLong Diamond Antifade Mounting Medium (Thermo Fisher Scientific, USA) and sealed with DPX (Phthalate Free) (Thermo Fisher Scientific, USA). The images corresponding to EMA1 and SSEA1 were analyzed using epifluorescence microscopy (Leica, Germany), and the images corresponding to DAZL and DDX4 were analyzed using confocal microscopy (Leica, Germany).

### Analysis of germ cell- and stem cell-specific markers by flowcytometry

cPGCs were labeled using primary antibodies including EMA1 (diluted 1:100; DSHB, USA) and SSEA1-conjugated AF647 (1:125 dilution; Santa Cruz, USA) (Fig. 1B-b). Anti-mouse IgM AF647 (diluted 1:500; JIR, USA) was used as a secondary antibody to label EMA1 primary antibody (Fig. 1B-b). For labeling the cells, 5 × 10^5^ cPGCs (either cultured in enriched or defined media) were counted and centrifuged. The cell pellet was then resuspended in 1 mL DPBS containing 5% FBS. After centrifugation, the supernatant was discarded, and cells were resuspended and incubated in 100 μL of each primary antibody for 1 h. The cells labeled with EMA1 were centrifuged and incubated with 100 μL of anti-mouse IgM AF647 secondary antibody for 30 min. 900 μL of DPBS containing 5% FBS was added to all tubes and centrifuged. The supernatant was discarded, and the cells were resuspended in 300 μL of DPBS containing 5% FBS. Control cells were labeled with only the anti-mouse IgM AF647 secondary antibody. All centrifugation steps were performed at 1200 rpm for 3 min. Flow cytometry analysis was performed using a BD device (BD Biosciences, USA).

### Analysis of pluripotency-related genes by RT-qPCR

The expression levels of *cNANOG* and *cPOU5F3/OCT4* transcripts were quantified using RT-qPCR. Total RNA was extracted from cPGCs using the RNA Isolation Kit (Qiagen, Germany), and 500 ng of RNA was reverse transcribed using the RevertAid First Strand cDNA Synthesis Kit (Thermo Fisher Scientific, USA) and random hexamer primers. qPCR was conducted with 50 ng cDNA in a 20 µL reaction with the RealQ Plus 2 × Master Mix Green (Ampliqon, Denmark) and an Applied-biosystem Real-Time PCR device (Thermo Fischer, USA) with three technical replicates. Primers that were used to amplify *cNANOG* (PCR product size: 183 bp) were 5′-GGGATTTATCTACCACAGAATGG-3′ and 5′-CACAGCCATGAACGGATA-3′. To amplify *cOCT4* (PCR product size: 87 bp) forward and reverse primers were 5′-ACGCTCTATGGGAAGATGTTC-3′ and 5′-CTTCAGCTTGCACATGTTCTTA-3′). The forward primer 5′-GAGAAGATGACACAGATC-3′ and reverse primer 5′-CAGAGTCCATCACAATAC-3′ were used to amplify *cACTB* (PCR product size: 118 bp). The PCR protocol consisted of an initial denaturation step at 94 $\mathbf{^\circ{\rm C} }$ for 15 min, followed by 45 cycles of denaturation at 94 $\mathbf{^\circ{\rm C} }$ for 30 s, annealing (at 50 $\mathbf{^\circ{\rm C} }$ for *cNANOG* and *cOCT4* and at 55 $\mathbf{^\circ{\rm C} }$ for *cACTB*) for 30 s, and extension at 72 $\mathbf{^\circ{\rm C} }$ for 20 s followed by a 10s extension at 72 $\mathbf{^\circ{\rm C} }$ for data collection. A melting curve analysis was performed between 60 and 95 $\mathbf{^\circ{\rm C} }$.

To find the most appropriate reaction temperature and the best concentration of primers, and to optimize the amplification and melting curves, different qPCR reactions were performed. Dilution series of cDNA was prepared to generate standard curves for *cNANOG, cOCT4*, and *ACTB* genes using the SYBR Green qPCR master mix (Ampliqon, Denmark). To this end, 2 uL of the cDNA from each dilution was added to 18 μL of the SYBR Green qPCR Mastermix in three technical replicates and subjected to real-time readings. To make a standard curve (Supplementary Fig. S3), the log_10_ of cDNA concentration for the *cNANOG*, *cOCT4*, and *ACTB* genes were plotted against the cycle threshold (Ct) numbers. We used the equation of E = − 1 + 10^(–1/slope) to calculate the reaction efficiency. The gene expression ratio for the *cNANOG* and *cOCT4* genes over the *ACTB* gene was calculated using the Pfaffl method^28^.

### Assessment of clonal expansion of cPGCs

The ability of media to support the clonal expansion of cPGCs was assessed. A single cPGC was picked up using a glass micro-needle under fluorescence microscopy and transferred into a single chamber of a 96-well plate, containing 100 µL of the enriched medium. The cells were fed every 2 days by replacing two-thirds of the medium until an appropriate number of cells was acquired (~ > 3 $\times$ 10^4^/each well of a 96-well plate).

### Plasmid resources and preparation

Transposon plasmids modified from the pBP vector (PiggyBac transposon vector) were generous gift from J. Silva laboratory (University of Cambridge, UK). DAZL and DDX4 promoter have been cloned by PCR amplification from chicken genomic DNA and cloned individually in the pBP vector (supplementary Fig. S4A-a, A-b, B-a, B-b). Transposase plasmid was modified from pCMV6-XL5 (Origene Technologies, Ref: pCMV6-XL5) where transposase gene came from Austin Smith laboratory (Institute for Stem Cell Research, University of Edinburgh, UK) and was cloned in the pCMV6-XL5.

### Transfection of cPGCs

To prepare the plasmid mix, 2 µg DDX4-tdTomato transposon (Supplementary Fig. S4A-a) and 2 µg *piggyBac* transposase were mixed with R buffer (provided in the NEON kit; Thermo Fisher Scientific, USA) in a final volume of 10 µL. To prepare the cell suspension, 1 × 10^6^ 120-day-old cPGCs were counted and pelleted. The cells were then washed with DPBS, pelleted again, and re-suspended in 10 µL of R buffer. To prepare the transfection mix, 10 µL of the plasmid mix was added to the 10 µL cell suspension. Using the NEON pipette and the pipette tip provided in the kit (Thermo Fisher Scientific, USA), 10 µL of the transfection mix was gently pipetted, and the tip was loaded into the NEON chamber. Electroporation was performed by applying 650, 850, or 1050 V for 50 milliseconds and 1 pulse. After the tip was unloaded from the NEON chamber, the cells were gently dispensed into a 24-well plate containing a pre-warmed enriched medium. The cells were incubated for 24 h and the medium was changed daily thereafter. To evaluate the efficiency of transfection, a portion of the cells was pelleted, washed twice with cold DPBS, and re-suspended in 200 µL of cold DPBS. The suspension was analyzed using flow cytometry.

### Promoter assay

cPGCs were transfected/electroporated by plasmids containing 2xHS4-DDX4-tdTomato (Supplementary Fig. S4A-a) or 2xHS4-DAZL-tdTomato (Supplementary Fig. S4B-a) to evaluate promoter functionality in the 120-day-old cPGCs.

### Gonadal migration assessment of cPGCs cultured in the enriched medium

Before injection, 2.5 d fertile eggs were placed vertically with the flattened end facing upwards and allowed to incubate for a minimum of one hour without rotation. The eggs were swabbed with 70% ethanol. A 30-mm circle window was opened on the flattened end of the egg using a small drill. Clonally-expanded/non-selected tdTomato-positive cPGCs, cultured in an enriched medium for two weeks, were successfully injected into the dorsal aorta of seventeen 2.5-day-old chicken embryos (at HH stage 14–16) in a volume of 5 µL containing 5 × 10^3^ cells. The window was sealed with Parafilm and the injected eggs were incubated at 37.7 ± 2 °C with 60% relative humidity and rocked through a 45° angle every hour until day 6 (at HH stage 26–28). The six-day-old gonads (at HH stage 26–28) were dissected using fine scissors and transferred to petri dishes containing cold DPBS. The presence of localized tdTomato-positive cPGCs was evaluated in all dissected gonads using a fluorescent stereo-microscope (Leica, Germany).

### Statistical test

For statistical analysis of data related to RT-qPCR, derivation rate, and expansion rate, the Mann–Whitney test was performed. Data were considered statistically significant at *p* < 0.05, and *p* < 0.01.

## Results

### Enriched medium improves derivation, expansion, long-term culture, and proliferation rates of cPGCs

The efficiency of derivation, expansion, long-term culture (Fig. 2B), and proliferation rates (Supplementary Fig. S2A, B, C) of cPGCs was compared between enriched and defined media by culturing cPGCs isolated from embryonated chicken eggs at HH stage 14–16. Three experimental groups were established for each medium, corresponding to HH stages 14, 15, and 16 (Fig. 2B). Judging by the number of derived and expanded cPGCs at each stage, a significant difference was not observed between the three stages of HH 14, 15, and 16.

At the end of day 21 (Fig. 2B-a), the confluent cPGCs (~ > 3 $\times$ 10^4^/each well of a 96-well plate) were individually transferred into the 48-well plates. Results showed that the derivation of cPGCs during days 14–21 of culture in the enriched medium was 1.75 times higher than that in the defined medium, regardless of the stage and sex of cPGCs. Similarly, expanded (Fig. 2B-b) and long-term cultured (Fig. 2B-c) cPGCs maintained in the enriched medium were 2.37 and 2.92 times higher than those in the defined medium at the end of days 28 and 56, respectively. The number of derived (Fig. 2B-f) and expanded (Fig. 2B-g) cPGCs in the enriched medium were significantly higher than those in the defined medium (*p* < 0.05).

We froze the long-term cultured cPGCs at the end of day 56. Three days later, two male and one female cPGCs were thawed from each experimental group for further proliferation (~ > day 120) (Fig. 2B-e). Evaluation of proliferation rates revealed that cPGCs cultured in the enriched medium had a shorter doubling time (DT) compared to those in the defined medium (29 to 34 h vs. 32 to 40 h, respectively) (Supplementary Fig. S2A, B, C).

For the rest of the experiments (including immunocytochemistry, flow cytometry, and qPCR), three highly-proliferated cPGCs from each group were used and named E-M14-1 (Enriched, Male, HH stage 14, number 1), E-F14 (Enriched, Female, HH stage 14), E-M15-2 (Enriched, Male, HH stage 15, number 2), D-F14 (Defined, Female, HH stage 14), D-M14-2 (Defined, Male, HH stage 14, number 2), and D-M15-1 (Defined, Male, HH stage 15, number 1) (Supplementary Fig. S2A, B, C). The clonal expansion capacity of cPGCs was demonstrated using the defined medium with FGF2 and ActivinA^9^. Our results showed that cPGCs cultured in the enriched medium were able to expand clonally (Supplementary Fig. S2D).

Long-term cultured cPGCs maintained in the enriched medium displayed prominent features including a high concentration of eccentric refractive granules (Fig. 2C-b; indicated by white arrows in the left inset compared to those in the right inset corresponding to Fig. 2C-d) and low dead cells (Fig. 2C-a, b, D-a, b, c; compared to Fig. 2D-d, e, f, and inset of Fig. 2C-c, d in which the red arrows indicate dead cells). Additionally, a high number of doublet cPGCs were observed in the enriched medium, indicating the healthy status of cPGCs which were actively dividing in long-term cultures (indicated by oval dash-lines in Fig. 2C-a, b, c).

### Enriched medium enhances the expression of stem-cell and germ-cell specific markers and transcription factors in cPGCs

**Figure 3:**
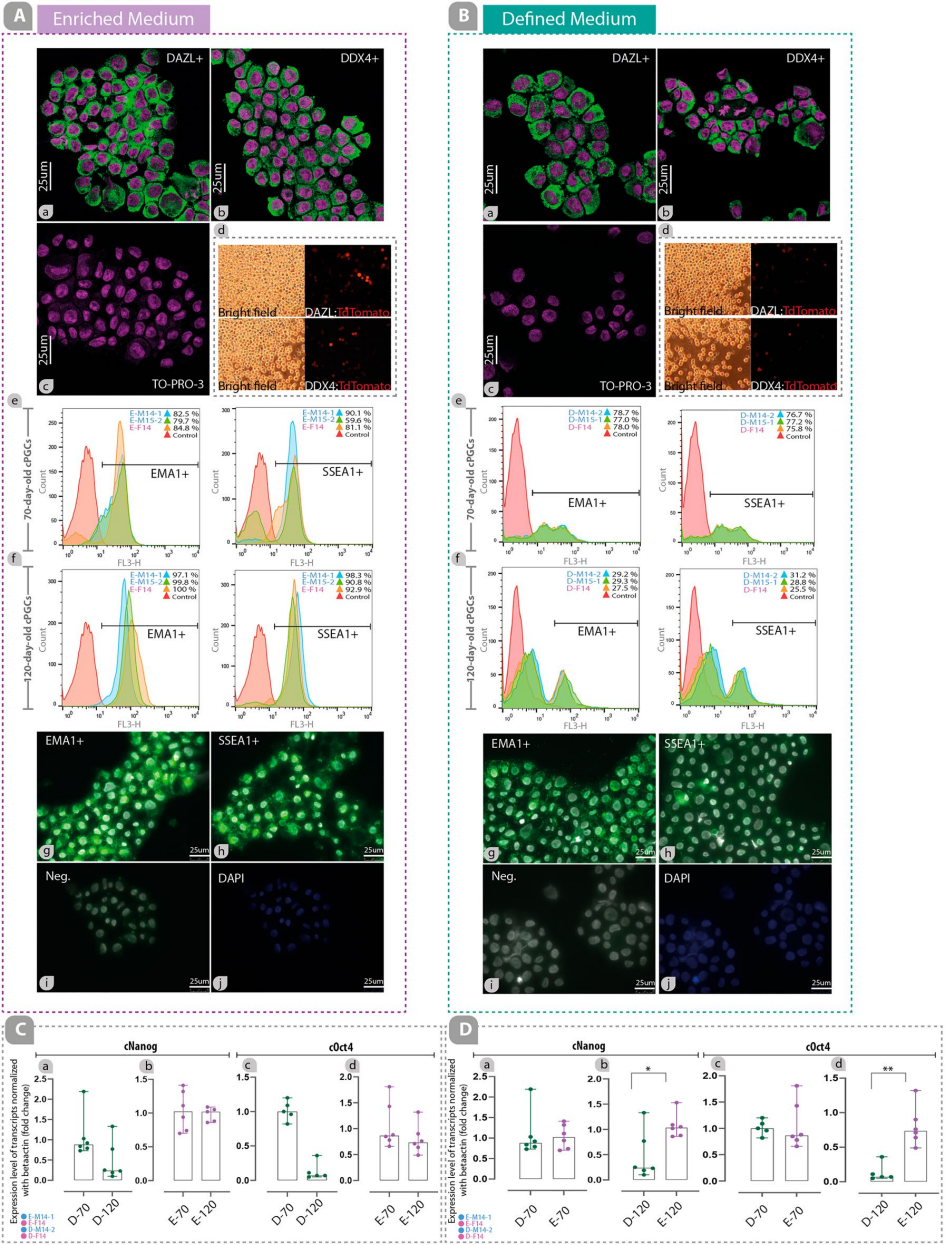
Enriched medium enhances the expression of stem-cell and germ-cell specific markers and transcription factors in cPGCs. DAZL-positive (**A**-a, **B**-a) and DDX4-positive (**A**-b, **B**-b) cPGCs, as well as unstained cPGCs (Ac, Bc; Negative control: it was only stained using secondary antibody) imaged by confocal microscopy are shown. The promoter assay shows the expression of tdTomato controlled by DAZL (**A**-d, **B**-d; top) and DDX4 (A-d, B-d; bottom) promoters in cPGCs. Panels **A**-e and **B**-e show the expression analysis of EMA1 and SSEA1 in cPGCs cultured in enriched and defined media by flow cytometry at day 70. Panels **A**-f and B-f show the expression analysis of EMA1 and SSEA1 in cPGCs cultured in enriched and defined media by flow cytometry at day 120. The cell membrane localization of EMA1 and SSEA1 on cPGCs are shown in panels **A**-g, **A**-h, **B**-g, and **B**-h. Also, no green fluorescence signals were observed in the negative groups (**A**-i and **B**-i). A DAPI staining was performed for each group (**A**-j, **B**-j). Panel **C** shows the comparison of the expression level of *cNANOG* and *cOCT4* transcripts between 70- and 120-day-old cPGCs cultured in defined (**C**-a, **C**-c) and enriched (**C**-b, **C**-d) media. Panel **D** shows the comparison of the expression level of *cNANOG* and *cOCT4* transcripts between cPGCs cultured in the defined and enriched media at day 70 (**D**-a, **D**-c) and 120 (**D**-b, **D**-d).

The germ cell developmental potency of cPGC established lines was evaluated through the expression of specific markers including DDX4 and DAZL (Fig. 1B-a). After long-term culture in both enriched and defined media, cPGCs were immunocytochemically positive for DAZL and DDX4 (Fig. 3A-a, A-b, B-a, B-b), as well as unstained cPGCs (Ac, Bc; Negative control: it was only stained using secondary antibody). The results of promoter assay driving the expression of tdTomato also indicated that DDX4 or DAZL promoters are active in these cells (Fig. 3A-d, B-d and Supplementary Fig. S2E).

The expression of EMA1 (a germ-cell specific marker) and SSEA1 (a stem-cell specific marker) in cPGCs cultured in enriched and defined media was evaluated by flow cytometry at 70 and 120 days (Fig. 3A-e, A-f, B-e, B-f). Three cPGC populations (two males and one female) were cultured in either enriched (E-M14-1, E-F14, E-M15-2) or defined (D-F14, D-M14-2, and D-M15-1) media for 70 days. Expression of SSEA1 and EMA1 was higher in cPGCs maintained in the enriched medium (Fig. 3A-e, A-f) compared to those in the defined medium (Fig. 3B-e, B-f). In addition, long-term culture from 70 to 120 days resulted in an increment of the SSEA1 and EMA1 expression in cPGCs maintained in the enriched medium (Fig. 3A-e, A-f), while a reduction of expression was observed in cPGCs maintained in the defined medium (Fig. 3B-e, B-f).

The expression of EMA1 and SSEA1 were also evaluated by immunocytochemistry (Fig. 3). The results of immunocytochemistry showed that long-term cultured cPGCs were positive for EMA1 and SSEA1 in both enriched (Fig. 3A-g, A-h) and defined (Fig. 3B-g, B-h) media. The expression of EMA1 and SSEA1 was detected in the membrane of the cPGCs, indicated by green fluorescence signals. No green fluorescence signals were observed in the negative groups (Negative control: it was only stained using a secondary antibody) (Fig. 3A-i, B-i). A DAPI staining was performed for each group (Fig. 3A-j, B-j).

The expression level of *cNANOG* and *cOCT4*, which indicate the stemness and pluripotency of cPGCs, were evaluated using qPCR at 70 and 120 days (Fig. 3C, D). The results showed that while the expression levels of these genes in cPGCs cultured in the defined medium (D-M14-2 and D-F14) decreased from 70 to 120 days (Fig. 3C-a, C–c), their levels in cPGCs maintained in the enriched medium (E-M14-1 and E-F14) remained almost constant (Fig. 3C-b, C-d). However, there was no difference in the expression levels of *cNANOG* in the 70-day-old cPGCs maintained in either defined or enriched media (Fig. 3D-a). Similarly, the type of medium did not influence the expression level of *cOCT4* in 70-day-old cPGCs (Fig. 3D-c). Remarkably, the expression levels of both genes were significantly higher in 120-day-old cPGCs maintained in the enriched medium compared to those in the defined medium (*p* value; 0.0411 for cNANOG and 0.0043 for *cOCT4*) (Fig. 3D-b, D-d). The amplification, melting, and standard curves for *cACTB*, *cNANOG*, and *cOCT4* can be found in the supplementary Fig. S3.

### cPGCs expanded in the enriched medium have the competence of homing and colonization in the embryonic gonads

**Figure 4:**
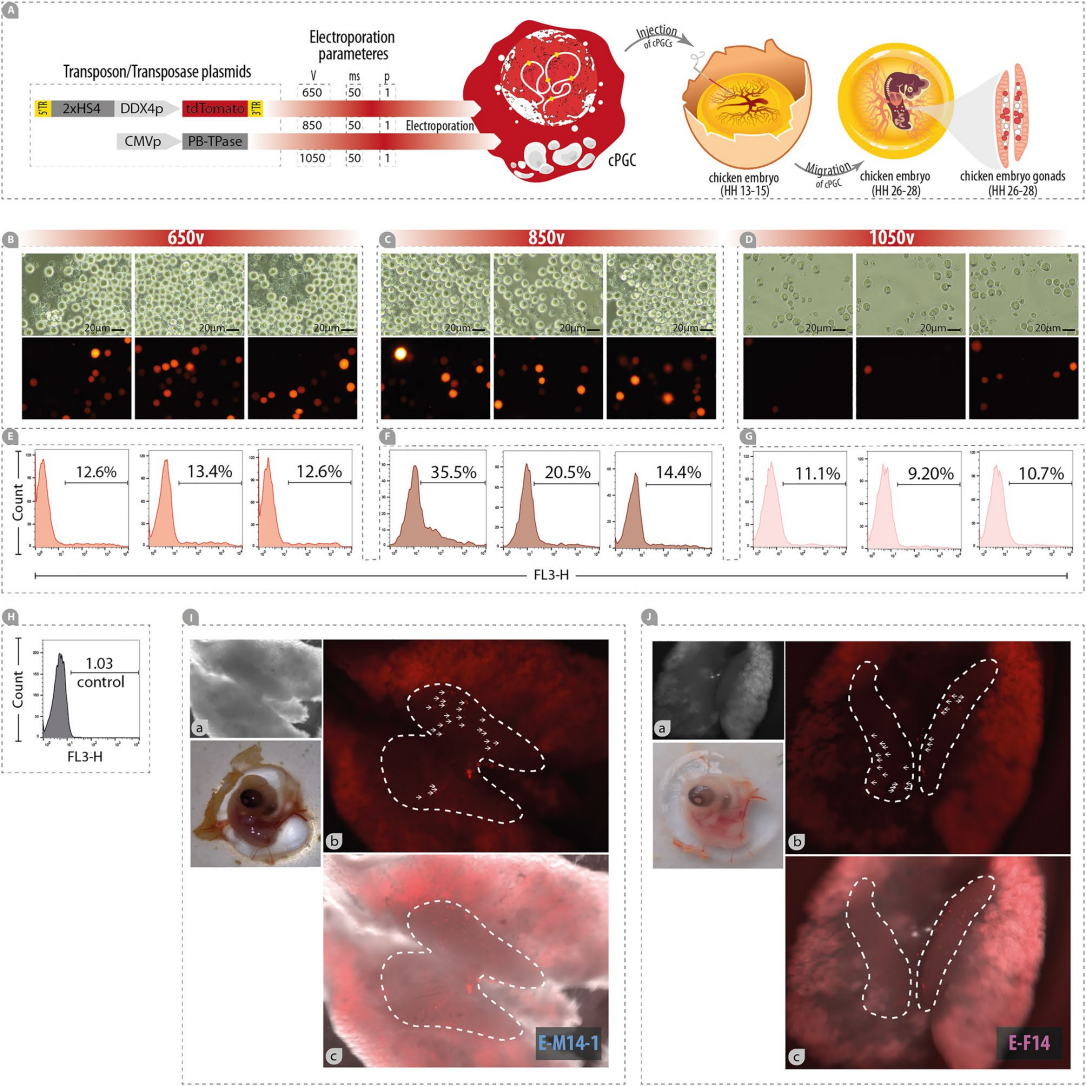
cPGCs expanded in the enriched medium have the competence of homing and colonization in the embryonic gonads. (**A**) schematic illustration showing co-electroporation of transposon/transposase vectors into the cPGC, injection of the td-Tomato-expressing cPGCs into the chicken embryo (HH stage 13–15), and localization of these cPGCs in the chicken gonads (HH stage 26–28). (**B**–**D**) The td-Tomato-expressing cPGCs that were electroporated in 650v, 850v, and 1050v, respectively. (**E**–**G**) Analysis of the electroporation efficiency by flow cytometry. H) Negative control (cells that were not electroporated). (**I**,**J**) Localization of td-Tomato-expressing cPGCs in the 6-day-old embryo gonads (HH stage 26–28). (**I**-a, **J**-a) Bight field images of dissected chicken embryo gonads (HH stage 26–28). (**I**-b, **J**-b) The td-Tomato-expressing cPGCs localized in the chicken embryo gonads (HH stage 26–28) imaged by red fluorescence. (**I**-c, **J**-c) Merged images of chicken embryo gonads at HH stage 26–28. v: volt, ms: millisecond, p: pulse.

To evaluate the migration potential of cPGCs cultivated in the enriched medium, reporter-expressing 120-day-old cPGCs were generated. To achieve this, the two lines of cPGCs (E-M14-1 and E-F14) were co-electroporated with a transposon system consisting 5′ITR-2XHS4-DDX4-tdTomato-3′ITR and the CMV-PB-TPase plasmids (Fig. 4A–D). The electroporation efficiency of cPGCs in the enriched medium were evaluated. The average electroporation efficiency was 12.86%, 23.46%, and 10.33% for 650, 850, and 1050 V (50 milliseconds, 1pulse), respectively, as determined by flow cytometry after 72 h post-electroporation (Fig. 4E–G). The cPGCs co-electroporated with 1050 V could not be recovered or divided after 72 h (data not shown) (Fig. 4D). However, both cPGCs co-electroporated with 650 and 850 V were successfully recovered and expanded (Fig. 4B,C). The clonally-expanded/non-selected cPGCs that carried the 2XHS4-DDX4-tdTomato in their genome were injected into the dorsal aorta of the chicken embryo at the HH stage 13–15. The results showed that tdTomato-positive cPGCs were localized in the embryonic gonads at the HH stage 26–28 (Fig. 4I,J).

## Discussion

In this study, we have found that an enriched medium containing KOSR not only preserves the expression of germ cell-related markers in cPGC lines, but also significantly enhances their self-renewal, pluripotency, and proliferation compared to the defined medium. In a preliminary study, we cultured cPGCs from embryonic blood in a defined medium. However, we observed a loss of propagation and morphological alterations in some cultures. This led us to hypothesize that these alterations may be due to the lack of some lipids, which may affect metabolism and pluripotency. Given the positive impact of lipid-rich albumin, an active ingredient in KOSR, on stem cells, we decided to test the rate of derivation, expansion, long-term culture, and proliferation of cPGCs in an enriched medium containing KOSR. We found that enriched medium improves derivation, expansion, long-term culture, and proliferation rates of cPGCs (Fig. 2). Also, enriched medium enhances the expression of stem-cell and germ-cell specific markers as well as pluripotency-related transcription factors in cPGCs (Fig. 3). Moreover, results showed that cPGCs expanded in the enriched medium are competent to home and colonize in embryonic gonads (Fig. 4).

The use of KOSR in the culture of murine and human primordial germ cell-like cells (PGCLCs) has been well documented. The induction medium containing 1% KOSR has been used to derive murine PGCLCs from pluripotent stem cells^29^. The generation of murine PGCLCs derived from embryonic stem cells/induced pluripotent stem cells (iPSCs) requires the use of ActivinA/bFGF/KOSR^30^. A defined medium containing KOSR has been used for the persistent expansion of human PGCLCs derived from human induced pluripotent stem cells^31^. KOSR has also been used in the generation of human PGCLCs from primed iPSCs^32^ or germ-line competent pluripotent stem cells^33^.

The fast propagation of cPGCs cultured in our defined medium in the first two weeks may have been due to the support of components of blood mixed with the medium. This is in line with a previous report^34^. However, the gradual loss of propagation or morphological alteration was observed in some cultures, severely affecting the derivation rates. We hypothesized that instability and loss of unspecified components of blood including lipids before the in vitro adaptation of cPGCs may result in inappropriate derivation and expansion. Cellular lipids can be supplied from the microenvironment or through de novo lipogenesis, which requires the production of essential fatty acids and cholesterols from carbohydrates^24^. Diminishing de novo lipogenesis and enhancing glutamine-dependent oxidative metabolism would be beneficial for reducing the metabolic burden^24,35^. The maintenance of FGF2-dependent ERK phosphorylation and the upregulation of gene expression in the MAPK pathway may be enhanced by lipid supplements^23^. Although the impact of lipids on cPGCs metabolism and germline features, compared to some growth factors, is unknown and might be involved in diverse bioprocesses, we reasoned that supplementing a lipid-rich mix could theoretically improve the developmental properties of cPGCs in vitro if lipids modulate metabolism, germline features, and pluripotency by reducing the burden of de novo lipogenesis.

The impacts of AlbuMAX, a lipid-rich albumin that is an active ingredient within KOSR, on stem cells have been demonstrated^25^. Given the constructive effect of AlbuMAX on stem cells^5,25^, we used an enriched medium containing lipid-rich albumin to cultivate the cPGCs.

In this study, we observed improved morphological features and a higher rate of derivation, expansion, and proliferation of cPGCs in the enriched medium compared to the defined medium. The cPGCs in the enriched medium retained their eccentric refractive granules. Also, there was a lower number of dead cells in these cultures for over 120 days. Although the exact composition of these granules has not been fully identified, glycogen is one of the prominent components found in their cytoplasm^36^. The presence of these granules in the cPGCs maintained in the enriched medium may be due to the use of lipid-rich albumin-containing KOSR, which shifts metabolism towards glutamine-dependent oxidative metabolism and reduces de novo lipogenesis^35^. In our study, the abundance of these granules was considered a sign of healthy and actively dividing cPGCs.

The derivation and expansion rates of cPGCs maintained in an enriched medium were almost two times higher than those in the defined medium (Fig. 2). A derivation rate of 12.5% of cPGCs cultured on feeder cells was previously reported by Lavoir et al.^2^. Whyte et al. reported that the derivation of cPGC in a defined medium containing Activin A was higher than in a medium containing BMP4^9^. The derivation rate of PGCs isolated from chicken or other birds was reported to be almost 60% using a defined medium^9,12^. In this study, the derivation rate of cPGCs cultured in the enriched and defined media was 68% and 40%, respectively. Additionally, 88% (8 cPGC lines out of 9 long-term cultured cPGC) of the established lines were expanded in the enriched medium, while 55% (5 cPGC lines out of 9 long-term cultured cPGC) were expanded in the defined medium. This is higher than what has been reported previously, where efficiency of line establishment of almost 50% was achieved^9,37^. However, all of these comparisons should be made with caution due to biological variations among the cPGCs isolated from different breeds. The improvement in derivation and line establishment of cPGCs cultured in enriched media may be due to genome consistency^5^ and the removal of extra-metabolic burden^24^. The enriched medium may provide a suitable microenvironment for enhancing the adaptation capacity of cPGCs from the derivation to the expansion stage. It should be noted that derivation rates may be dependent on strain and sex, and the quality of each component of the medium may also affect the derivation rate of cPGCs. Therefore, a comparison of derivation rates among different studies may not be logical.

cPGCs cultivated in the enriched medium displayed a faster proliferation rate compared to those cultivated in the defined medium, with a DT of 29 to 34 h vs. 32 to 40 h, respectively (Supplementary Fig. S2A, B, C). This finding is in line with the study by Zhong et al.^5^, which showed that murine ESCs cultured in a defined 2i/LIF medium supplemented with AlbuMAX proliferated more rapidly than those cultured in defined 2i/LIF medium alone. Zhong et al. also demonstrated that 2i/LIF medium supplemented with AlbuMAX can efficiently prevent X chromosome loss in female murine embryonic stem cells. The authors attributed this to the stimulation of lipid-induced Erk2 activity, which improves genome stability in murine ESCs during long-term culture. AlbuMAX also promotes nucleotide and Acyl-CoA biosynthesis and maintains telomere length, resulting in a normal karyotype in both sexes of murine ESCs.

In our study, we found that the rate of 120-day-old female cPGCs maintained in the enriched medium was higher than those maintained in the defined medium. This may be due to the maintenance of genome stability and normal karyotype in cPGCs cultivated in the enriched medium over a long-term time. Previous studies have also reported improved derivation rates for female cPGCs using a medium supplemented with ovotransferrin^37^ and higher in vitro culture efficiency for both male and female cPGCs cultured in the medium supplemented with chicken serum instead of OT^9,12,37^. It seems that the derivation, expansion, and long-term culture of cPGCs may depend on the quality of the materials, breeds from which the cPGCs derived, and combination of components which are essential for cPGC survival. In conclusion, our results suggest that the rate of cPGCs derivation and line establishment using enriched medium surpasses that of other previously used media.

Generally, female cPGCs cultured on the feeder layer tend to form tight clustering, resulting in slower dividing compared to the male cPGCs^34^. This phenomenon was not observed in cPGCs cultured in chemically-defined media^9,12^. In contrary to chemically-defined media^9,12^, we observed tight clustering both in male and female cPGCs cultured in defined media. It may be due to the intrisic features of the cPGCs derived from JA57 breed. It has been reported that female cPGCs cultured in a defined medium have DT comparable to male cPGCs^9,12^, a finding that follows our observations of similar proliferation rates for both male and female cPGCs cultured in either enriched or defined media (Supplementary Fig. S2A, B, C).

The germ-cell developmental potency and the markers related to pluripotency of cPGCs cultured in the enriched medium were significantly improved compared to those cultured in the defined medium (Fig. 3). Some specific lipids have been suggested to play a critical role in establishing pluripotency^27^ and germ-cell developmental potency^38–40^. For instance, the addition of AlbuMAX to 2i/LIF defined medium has been demonstrated to significantly upregulate and improve the expression of pluripotency genes in murine ESCs^5^. It has been demonstrated that KOSR supports the steady growth rate of spermatogonial stem cells (SSCs), the expression of SSC markers, and the continuous growth of SSCs in vitro^40^. Also, The lipid and fatty acid-enriched conditions can enhance cell proliferation and improve reprogramming efficiency by increasing cAMP levels^27^. Based on these findings, we hypothesized that maintaining germ cell developmental potency and pluripotency could be advantageous for the long-term culture of cPGCs. Transcription factors such as *NANOG* and *OCT4* are vital for the survival and formation of PGCLCs^41,42^. Pluripotency gene networks including *NANOG*, *OCT4*, and *SOX2* must be active in unipotent PGCs before specification^30^. *OCT4* loss triggers apoptosis of PGCs^41^ and loss of *NANOG* function significantly impairs PGCLC specification^42^. Lipid-rich albumin in KOSR may improve *cNANOG* and *cOCT4* expression for cPGCs cultured in the enriched medium, leading to robust and actively dividing cells.

Signaling factors such as activin A and FGF2 have been found to generate intermediary germline-competent murine epiblast-like cells. These intermediary cells can be successfully transformed into PGCLCs when exposed to a combination of growth factors and 1% KOSR^43^. It has also been shown that naïve murine ESCs cultured in bFGF/ActivinA media acquire the competence for PGC-like fate^44^. Based on these findings, activin A and FGF2 were used to maintain consistent germ cell developmental potency in long-term cultured cPGCs, as steady germ cell developmental potency could enhance the efficient migration and contribution of cPGCs to both male and female gametes^9^. In this study, the expression of germ cell-related markers including SSEA1 and EMA1 was evaluated in cPGCs cultured in both enriched and defined media at two time points (70-day-old and 120-day-old cPGCs). Results showed that the expression of both markers increased in cPGCs cultured in the enriched medium compared to those cultured in the defined medium from day 70 to 120. DAZL and DDX4 were efficiently expressed in both culture conditions, as indicated by immunocytochemistry and promoter assay, even in 120-day-old cPGCs.

We evaluated the migration ability of E-M14-1 and E-F14 (harboring 2XHS4-DDX4-tdTomamto) into the developing gonads of embryos (Fig. 4). Both lines demonstrated the ability to migrate from peripheral blood into the gonads of the embryos, indicating that their germ cell potency has been preserved. This finding is along with the findings of previous studies, in which established lines of cPGCs maintain their ability to localize in the embryonic gonads^9,12^.

## Conclusions

Three methods have been introduced for the culture of cPGCs. These include feeder-dependent culture in an undefined medium, culture in the defined medium containing OT, and culture in the defined medium containing CS. Here, we show that culture in the defined medium containing KOSR can significantly enhance the expression of pluripotency genes, increase proliferation rate, and support germ cell features. The in vivo development of cPGCs has unique cellular requirements and a delicate balance of signaling pathways and transcription factors that regulate the expression of pluripotency and germ cell-related genes (Fig. 1). These pathways must be activated consistently to ensure the normal development of cPGCs towards germline specification. Although some of these pathways have been identified in cPGCs (Fig. 1A), maintaining their optimal function in vitro can be challenging. Further research is needed to clarify differences in the effects of KOSR, OT, CS, or any combination of these components on the growth and development of cPGCs. For example, one area of research would be to elucidate lipid-related pathways which are functional in cPGCs.

## Supplementary Information

Supplementary Figures.

## Data Availability

All data generated or analyzed during this study are included in this published article (and its supplementary information files). Also, All data are available from the corresponding authors upon reasonable request.

## Abbreviations

cPGCs: Chicken primordial germ cells
E: Enriched medium
D: Defined medium
HH: Hamburger–Hamilton
KOSR: Knock-Out Serum Replacement
OT: OvoTransferrin
ITS: Insulin-Transferrin-Selenium
FBS: Fetal bovine serum
CS: Chicken serum
ESCs: Murine embryonic stem cells
NEAA: Non-Essential Amino Acid
Pen/Strep: Penicillin/Streptomycin
DPBS: Dulbecco’s phosphate-buffered saline
BSA: Bovine serum albumin
IgG: Immunoglobulin G
IgM: Immunoglobulin M
FITC: Fluorescein isothiocyanate
AF647: Alexa Fluor 647
DT: Doubling time
PGCLCs: Primordial germ cell-like cells
iPSCs: Induced pluripotent stem cells
SSCs: Spermatogonial stem cells
