# Supplementary material for: Enhanced cultivation of chicken primordial germ cells
Source: Sci Rep. 2023 Jul 29;13:12323. doi: 10.1038/s41598-023-39536-1 (PMC10387062; doi:10.1038/s41598-023-39536-1)
Supplement: Supplementary file 1 — Supplementary Figures. [file 41598_2023_39536_MOESM1_ESM.pdf]

Supplementary information for

Enhanced Cultivation of Chicken Primordial Germ Cells

Nima Dehdilani [1,¥], Sara Yousefi Taemeh [1,¥] , Sylvie Rival-Gervier [2], Guillaume Montillet [2], Clémence Kress [2], Christian Jean [2], Lena Goshayeshi [1], Hesam Dehghani [1, 3, 4, *], Bertrand Pain [2, *]

[1] *Stem Cell Biology and Regenerative Medicine Research Group, Research Institute of Biotechnology, Ferdowsi University of Mashhad, Mashhad, Iran.*

[2] *Univ Lyon, Université Lyon 1, INSERM, INRAE, Stem Cell and Brain Research Institute, U1208, USC1361, Bron, France*

[3] *Division of Biotechnology, Faculty of Veterinary Medicine, Ferdowsi University of Mashhad, Mashhad, Iran.*

[4] *Department of Basic Sciences, Faculty of Veterinary Medicine, Ferdowsi University of Mashhad, Mashhad, Iran.*

¥ Co-first authors

* Co-corresponding authors: dehghani@um.ac.ir, bertrand.pain@inserm.fr

This file contains 4 supplementary figures.

Supplementary Figure S1. Procedures of taking the chicken embryonic blood from 2.5-day-old chicken embryo for cPGCs isolation. A) A 30-mm circle window was opened on the flattened end of the egg using a small drill. B) The cut circle egg shell was removed. C) Opened window was sealed with PARAFILM. D, E) The 2.5-day-old chicken embryo (stage 14-16HH) was seen after removal of egg shell, outer shell membrane, and inner shell membrane (Heart beating and prominent dorsal aorta are the sign of healthy embryo indicated by white arrow and white arrowhead). F, G) A Glass microneedle was used to take blood from the dorsal aorta.

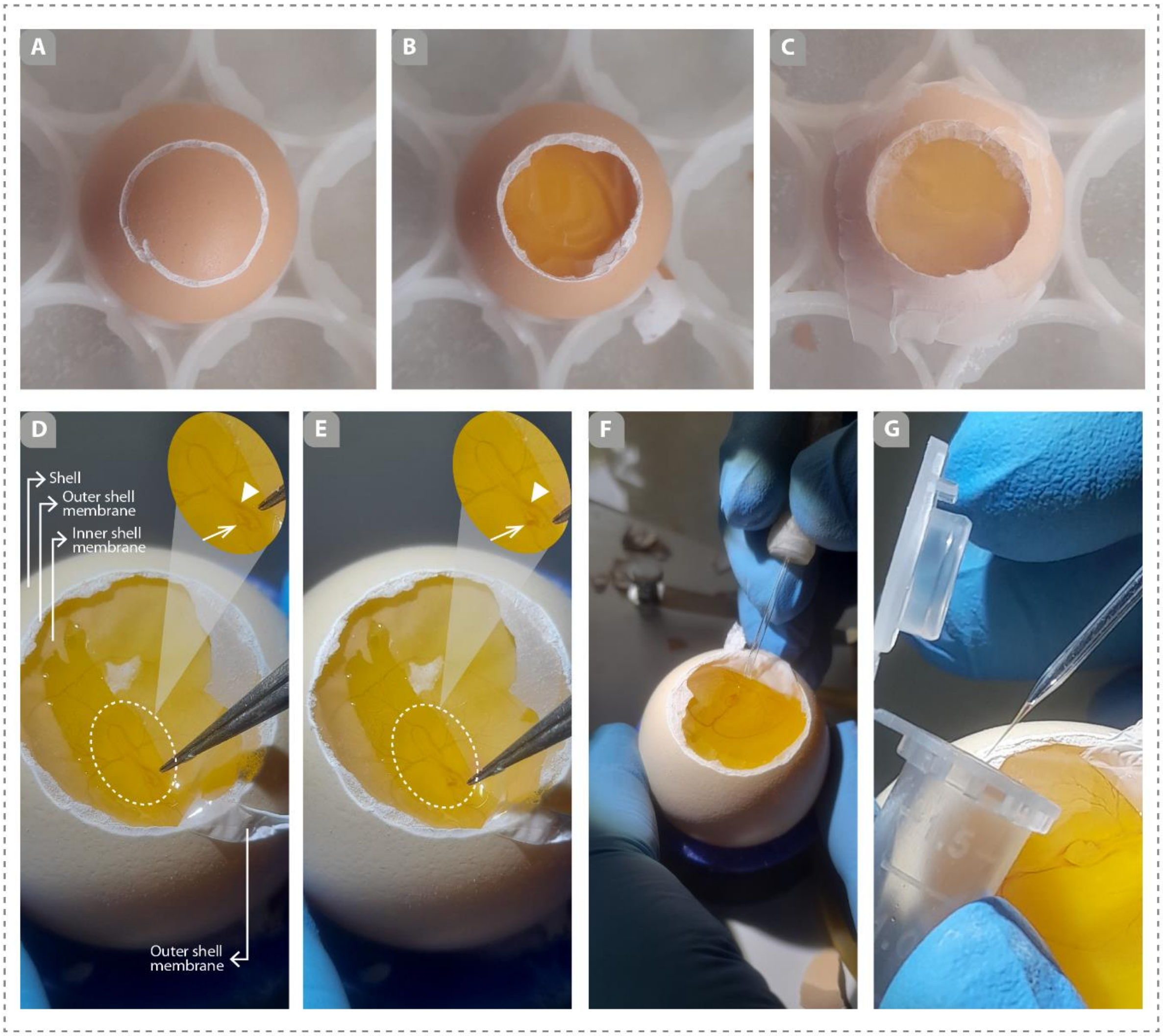

Supplementary Figure S2. Proliferation and promoter assay for cPGCs cultured in enriched and defined media. A, B, C) Doubling time was evaluated for two male and one female cPGC in three different embryonic stages in enriched and defined media from day 0 to 10. D) Clonal expansion (bottom panel) of a single cPGC (top panel). E-a,b,c,d) 120-day-old tdTomato-positive cPGCs driven by DAZL and DDX4. E-e,f,g,h) EGFP-positive and tdTomato-negative DF1 cells driven by CMV and DAZL/DDX4, respectively; DF1 cell lines were transfected using Lipofectamine 3000.

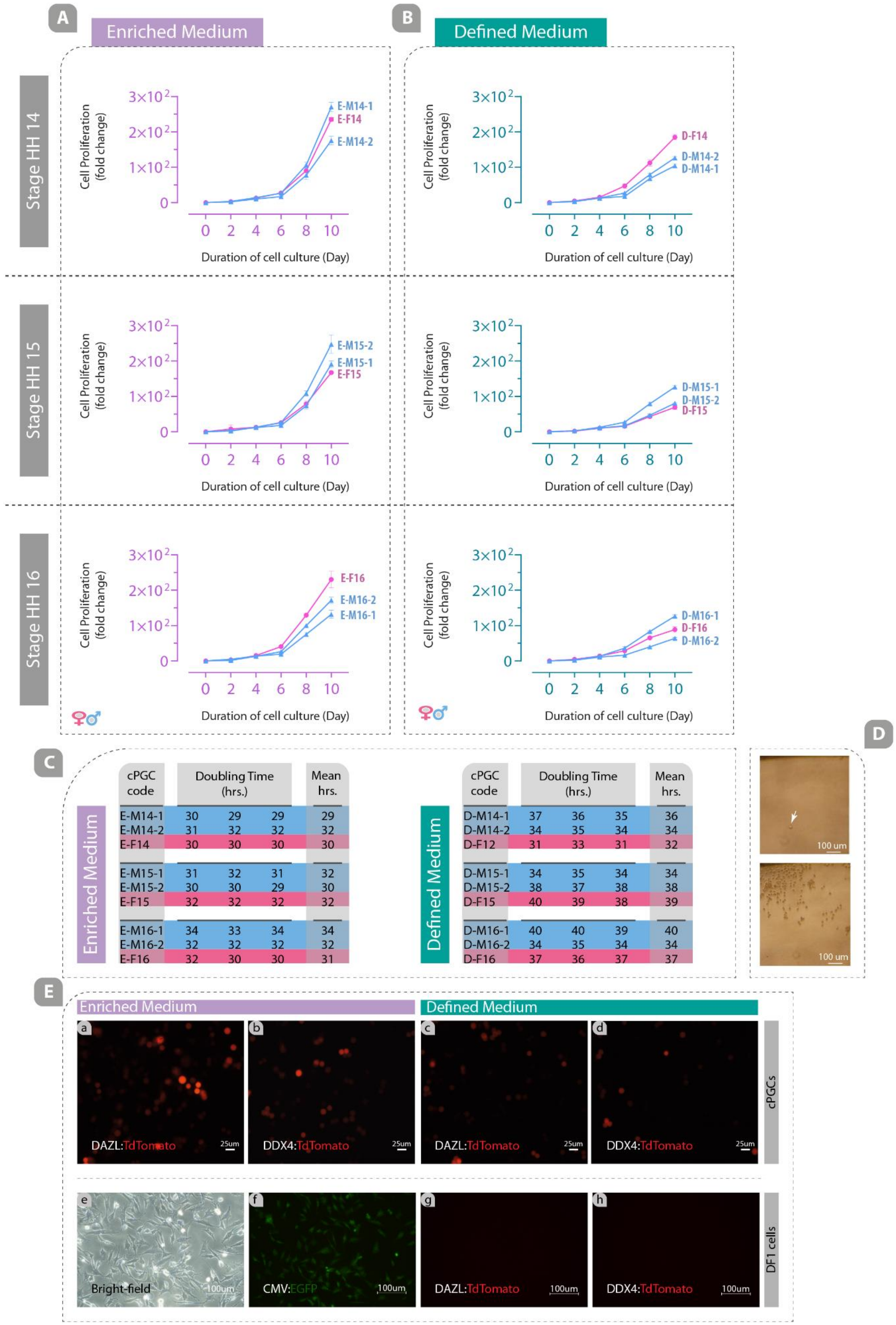

Supplementary Figure S3. Amplification, melting, and standard curves for *cACTB* , *cNANOG* , and *cOCT4* transcripts. Amplification, melting, and standard curves are illustrated for *cACTB* (A), *cNANOG* (B), and *cOCT4* transcripts. To make a standard curve, the $\log_{10}$ of cDNA concentration for *cNANOG*, *cOCT4*, and *ACTB* genes were plotted against the cycle threshold (Ct) numbers. Based on the equation of E = -1 + 10^(-1/slope) and standard curve equation formula (y= -3.5749x + 64.1815 for *cACTB*, y= -3.5500x + 64.8196 for *cNANOG*, and y= -3.6130x + 64.3100 for cOCT4), the efficiency of each reaction was calculated. Gene expression ratio for *cNANOG* and *cOCT4* genes over *ACTB* gene was calculated using the Pfaffl method of relative quantification.

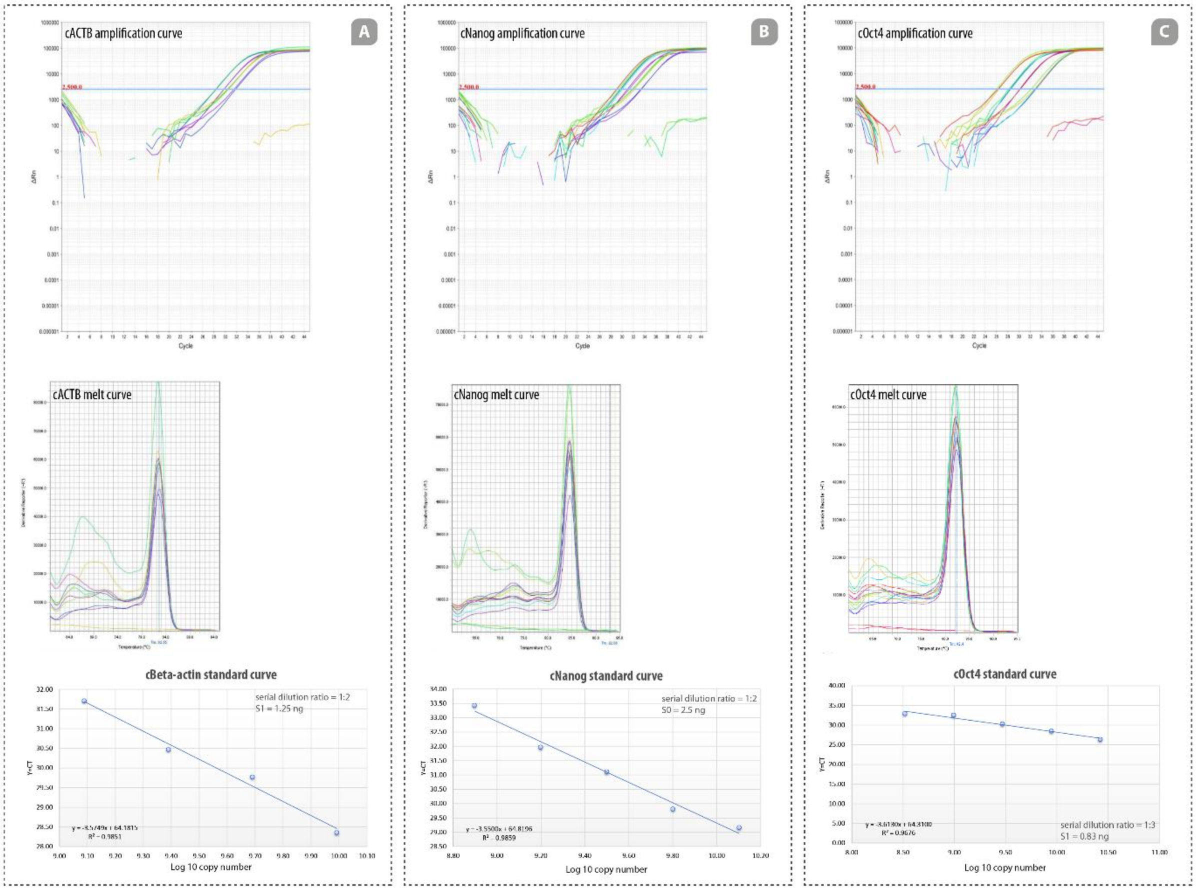

Supplementary Figure S4. Sequences of DDX4 and DAZL promoters. A-a) The transposon cassette containing DDX4 promoter. A-b) Sequence of DDX4 promoter. B-a) The transposon cassette containing DAZL promoter. B-b) Sequence of DAZL promoter.

A

a

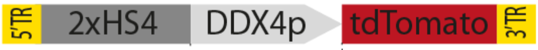

b **Sequence of DDX4 promoter**

GCTCACAGAAAATCTCTAGTTGGTAAGGTTACCTTTTTTTTTGTTGTCGCTCCCAGTTAAACCAAGGTGGGAACACATCCTTTTCATAGTTGTATTGTAGGGATGTTTA
TAGCTATTGCTAGGGAAAACTGAACAGCGTGCAAGGAAGTCAGCACTGACACAGCTTTCCCACGTGAGAGAGCTATTTCAAAGCAAGATCAGCACATATCCCAATC
TGTACTTCCTCAAACACAGCCCAAAACCGATAGCAAGGCCAAGCAGCAGCACTGCTCCTTCAAGGAAACTGCTGCATAATCTGAATTTCAGACGGCAGGAGGAAA
CAAGGCAGCAGACTGATGACCTACCACCCTGAGTCTCAGCTACATGCGATCCGAGCCACTCCAACTCTGCTTCCTGCAGCCCTTCCTTCACGCTCATCTTTTGCACCT
TAGGCACTCTTATAATTCAAGTACTTTGTGGCTTTGGGATATTTGAAGAGCTTGGTCAGTTAGTCACAAGTCTGGCCACGTGCTATCATATTAGTTTGAAAAGCAATG
GAGACACCATCTGCTGATGCTCAAAGTGGTTACAACCAAAACACAAAAAAGCAGAGCTGTGGGAAGAATTCAACATTTTGATTATGCAAGAAGCTAGTCCCAGCCT
TGAAATCCACCATCTGCATCATGAAAGACCTAAGTAGTTAAAGCCACAGCAGACATACAGCTTCTATTTCCTTACCTTCTTCATCATTAACTACAGGTCTTGGAAGATT
TTTGCTGGGAAAAAGCTTTTATTGCAAGAACTGTAATTTATTAACAGGGAAACATGAAATAAATGTGTAAATTCTCCTGCACTCCCACTGTCATTAAAAACGGCTTTA
AGAAAGAGTATTCAGTAACTGCCTGCATTGTGTTGTGACTTTCTACTCTGTGACACACAGCCTTCTGGGCAAAGCACATATTCTGCCATGCATGTGGGTCGTGCCTTG
GAAAATGGGAACCCACATTCACTGAGGGCCTCTTGATGAGCTTTCCCCTTGAGAACAGCGAGGTCTCATGGATATCTTCTCTTCTCCAAGCCAAATAAGCCCAGATC
CCTCAGCTTTCCTTCATGGAGAGGCTCCAGCCCTCTGATCATGCCTGCAGCCCTTCTCTGGACCTGCTCCAGCAGCCCCACACGCTCCTCATGCTGGGACCCCAGAT
CTGGACATGGTATTGCACATGGGGCCTCACAACGGCAGAGCTGAGAGGGACAATTCCCTCCCTCAGCCTTTAATTCTGCACGTACTTAATTTTGTCTGTATTTTTTTT
GCAATAGAATAGCCTGCATGCTAGTTGCTGTGTCGCACAGCTGAATTCACTAGGTTCCTGTGAAACAAAGGGCAATCCCACAGCCACTGTAGCACGTGAGGAGCCA
CACAGGTGCTCTAATTCCTACAGGACAGGCCTAGGGACAGAGCGGCCCTAGGACTGCCCTCCCGCACCTCCTAGCTTCACCTTTTGCCCGCGATCTTTAAGTACCCT
GAAGCATAAACAGGGAAAGCGCCGCCCGCAGCCTCACCCTGCTTCCCGCCCAACGCGGCACCGCCCAGGCCGCAGCCGCCCACTAAGAGCACTAGCGCCACCTT
CTCACCCCACCCCCACCACGCGTTCCTAGCGGCCCTGAGAGCTGCTGCGCATGCGCCGCCTGACGATTCGCCCTCCCATTGGCTGGCGGTCGAAGCACGGCGGGG
GCACGCGGTGGCGGCTATATAAGGCGTCTCGGAAGACGGCGCCATGCTATTTGGAGCGGAGAGTGAAAGTTACAGTTCCTGGTGCTGGTAGGGAGTGTGGCGCG
GAGCGGAGCGCTGCGGCTCATCGGAACCACAATGGAGCCATAGCAGAGCCGGGCGTGGGGGCAAGGGCAGTGCGTGCTGGGGAGGGCTCCGTCGCGTGGCCA
CGTCGCGAGAGCCGTCGGGATGGTGGCGTCAGGGCGGGGGGTGCTGCTAACGTGCTCCTGGTCCTGCAGGTGGGCTGCTGGCATT

B

a

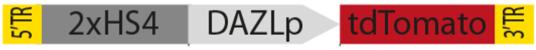

b **Sequence of DDX4 promoter**

GGCCAAATAAAGTAACATGTGAGAAAACAGCTTGCTTGTTATATGCACTCCAGTTGATCAGTTTAACTTTGTACTCTTTTTTTTCCCCAGATTAATCAGTCTTATACCTT
TGAAATTTATAGAAAGTTGCTAAGCTAGCACAGTCAAGAGTTTGTCACAGCATCCATAATGTTCCCTTCCCCAGAAGGGTCTGAAAATCTAGATATATTCTCAAGAGA
AGAAAACAAAAAGTATTTAAAACCAGCTTCCAAAGGCAGTGGAAGCACCTTATTAACATCGAAAAAGAAAAGCTTCCAGAGATATGTCCCTTCTAGAAAGATCTTA
ACAGTCTAGTGAGAAGGTGTTAGAAAAGGCATTATGGTGTAATGGAACCAGCCTGCTCCAGAGTATCCATATAACATCGGTGTGAGAAAACATTGCCAAAACGTTCA
GCTTTGCCTCAAAGTGGGCAACTCTAACCAAAGCCTCCTCAAGCAGTACAACCTCACTGGGCATATAACGCGGCTCTTCTGACAGGAAGGTGCTACCACTACGCTCT
CTGAACAGCAGGGCCTCAAACAATGGCCCTACCTAATCTCCTAACAAAGGAAAAGGTTACGGCACACATCGCCCTTCGTCTTCAGCCCTCTCCTGAAACACACACC
GAGGGCTGAACTCTCCCACACCGCGGCGGTGCGGTGCGGTGCGGTGCGGGCCGGACCAGGCCAGGCCAGGCCAGGCCAGCGGGGCGGGTGGGGCTTTCCCCG
CCCCTTCCCCCGGGGTCTACTTAAGAAGAGCGGCCGTCGAAAAGAACTGCCTCTTTCGCACCCCTCTTAAAAAAGAAAAGAAAGAAAAAAAAAAAAAAAGACAAA
AAAAATACAAACACAAAAAAGTGGGGTTCTTTAGTGTCTGTTTTCCCAACACTCCAATTGTTTTTGTCTTGAAGGCCTCGTTTGTTTTTAAGTGTGCGGGCGCTGTC
ACAGCTCCGGGGAACGATGGTAAGTCGGGACCGGGGACAACGGGGGAGCGGAGCGGTGGTCGGGCGGGTGGAGAGCAGGGTGTGAGGTGCGCCGGGCCGG
CGGGCAGTTGGCGGGGCCTCCTGAAGCTGCGGGAGTCCCGCAGCTGCGGCGCTGCCGCCTCACAGCGCCTTGCGGAGACTCTTGAGAATTACTCCCTTTTACAAT
AGGTTCTAGAATCTTACGTGTACGGTATGAATTTCAGTGATACTGCGAGAAAATGATAAGAAAAGAAGGCTGAGACATGAGTTGGTGTTGTGGTCAGAGGCGTGGT
GTCAGGAGGACTTCGAGGCAGGAGACGCGTCGTGCCGCGAGCGGCCATTTTGTGACTCCGGGCACAGTCACGGCCCTTCTACTCCGCCGCCTCCGGTCGGTGCC
CCGAGGTGGCAGTTGCACCCGCTCGGCGCTGGAGCCCGGCGTGACGCCTTGCCCTGCGACCGCGGGCTCTCCATCAGCTCCGTCTATTCTCAGCCTTTTGGCACTC
CCTCCCCTTTCCCTTTTCCTAAGCCCGTCCAGGCCCGTGACCCGTTTCCCGGCGCCGAGCTGTTGAGCCGCCCGAGCGGACCGTTATCACCTCGGGAGCTCCGGCC
CCACCAGGCGGTTTCCGGCAGCGCTCCGCGGGCCCCCAGCTGTGGTCAGCGGGGATGCTGTCCGGCCCCGCGCGTGCCCCTTCCATGCAAAGAGACCTTCGTCTT
CGAGAGTTTTCTGCGTAATGTGGTCCCCGTTGGGGCGAAGCCTCGCGTCGCTGGGTGCGGGTACAGTTCTGGTTGGTGCTAAACGTGTGCAGGGAAGGGAGCGG
TAAAGCGTTCCTGTCGAGGTGACTGTGCTCTGGTACACGAGCCGGCAGCTGAAACTAAACTGGTTGCACACAGTACTGTTTCTCAAGAAATTCTTTATTTTCGGGTT
TTTTTTACGCACGGTATCTGCTATTCTAACGCTGACACCTCAGCAACTTCCAGGCAGGACCGGGTAGT
